# Comparative analysis of liver involvement caused by two DENV-2 lineages using an immunocompetent murine model

**DOI:** 10.1038/s41598-021-88502-2

**Published:** 2021-05-06

**Authors:** Fernanda Cunha Jácome, Gabriela Cardoso Caldas, Arthur da Costa Rasinhas, Ana Luisa Teixeira de Almeida, Daniel Dias Coutinho de Souza, Amanda Carlos Paulino, Raphael Leonardo, Ortrud Monika Barth, Flavia Barreto dos Santos, Débora Ferreira Barreto-Vieira

**Affiliations:** 1 Laboratory of Viral Morphology and Morphogenesis, Instituto Oswaldo Cruz, Fiocruz, Avenida Brasil 4365, Rio de Janeiro, RJ 21040-900 Brazil; 2 Laboratory of Viral Immunology, Instituto Oswaldo Cruz, Fiocruz, Avenida Brasil, 4365, Rio de Janeiro, RJ 21040-900 Brazil

**Keywords:** Microbiology, Diseases

## Abstract

Dengue (DEN) is the most prevalent arbovirus among humans, and four billion people live at risk of infection. The clinical manifestations of DEN are variable, and the disease may present subclinically or asymptomatically. A quarter of patients develop classical dengue (CD) or severe dengue (SD), which is potentially lethal and involves vascular permeability changes, severe hemorrhage and organ damage. The involvement of the liver is a fairly common feature in DEN, and alterations range from asymptomatic elevation of transaminases to acute liver failure. Since its introduction in Brazil in 1990, two strains of Dengue virus (DENV) serotype 2 (DENV-2) have been detected: Lineage I, which is responsible for an outbreak in 1991, and Lineage II, which caused an epidemic greater than the previous one and had a different epidemiological profile. To date, studies on different strains of the same serotype/genotype and their association with disease severity are scarce. In addition, one of the greatest challenges regarding the study of DEN pathogenesis and the development of drug and vaccine therapies is the absence of an animal model that reproduces the disease as it occurs in humans. The main goals of this study were to assess BALB/c mouse susceptibility experimentally infected by two distinct DENV-2 strains and characterize possible differences in the clinical signs and alterations induced in the liver resulting from those infections. Mice infected by the two DENV-2 lineages gained less weight than uninfected mice; however, their livers were slightly heavier. Increased AST and AST levels were observed in infected mice, and the number of platelets increased in the first 72 h of infection and subsequently decreased. Mice infected with both lineages presented leukocytosis but at different times of infection. The histopathological changes induced by both lineages were similar and comparable to the changes observed in DEN fatal cases. The viral genome was detected in two liver samples. The results demonstrate the susceptibility of BALB/c mice to both DENV-2 lineages and suggest that the changes induced by those strains are similar, although for some parameters, they are manifested at different times of infection.

## Introduction

DEN is considered the most important arboviral disease in the world and classified by the World Health Organization (WHO) as the vector-borne viral disease with the fastest dispersal; thus, it has enormous potential to cause major epidemics worldwide^1,2^. The disease is currently endemic in over 125 countries, and global estimates vary. While a study suggests that approximately 50 to 200 million people are infected annually, with 500,000 episodes of SD and more than 20,000 deaths related to the disease^3^, others estimate that approximately four billion people are at risk of infection and 390 million are infected each year^4,5^.

DEN’s etiological agent is the dengue virus (DENV), an arbovirus belonging to the genus *Flavivirus*, family Flaviviridae. DENV presents four distinct but antigenically related serotypes, DENV-1, -2, -3 and -4, and is maintained in nature by a transmission cycle involving vertebrate hosts and blood-sucking mosquitoes of the genus *Aedes*, with humans being the only host capable of developing clinical forms of infection^6^. The viral particles are enveloped and spherical, present an icosahedral nucleocapsid and measure approximately 50 nm in diameter, and the viral genome consists of a single-stranded positive RNA of approximately 11,000 bases^7^, which encodes three structural proteins (E [envelope protein], M [membrane protein], C [core protein]), that constitute the viral particle and seven nonstructural (NS) proteins (NS1, NS2A, NS2B, NS3, NS4A, NS4B, NS5) that are expressed in infected cells^8,9^.

DEN presents a wide spectrum of clinical manifestations with unpredictable evolution, and different organs are involved^10^. In addition to manifesting classic dengue symptoms, the disease can present different clinical forms, including asymptomatic cases, undifferentiated febrile illness, or severe hemorrhagic disease, such as DHF and DSS^11^. Observations of a set of clinical and laboratory parameters have led to the early identification of severe cases, and the WHO^12^ classifies DEN as dengue with or without warning signs and indicates that SD is a potentially fatal outcome involving plasma leakage, fluid accumulation, respiratory distress, severe bleeding, and organ impairment. The factors leading to the different DEN manifestations are yet to be understood. However, severe forms of the disease are often associated with secondary infections, the DENV serotype, viral strain virulence and host genetic factors^13^.

Liver involvement is a fairly common feature in DENV infection^14,15^ and may be a direct effect of viruses infecting cells because hepatocytes and Kupffer cells are susceptible to DENV^14,16,17^ or it may represent an immune response against the virus because after recognizing DENV particles, Kupffer cells and macrophages release cytokines that activate inflammatory cells^18–20^. The tissue alterations caused by the infection range from an asymptomatic increase in transaminases to acute liver failure, which has fatal outcomes^21–23^. The most commonly reported clinical alteration is the elevation of ALT and AST levels, which are observed in the initial days after onset of fever and peak in the period of convalescence^18,22^. Hepatomegaly is common as well; however, it is more frequently observed in patients with DHF^15,24,25^.

Studies carried out with mouse models, HepG2 and Huh7 hepatoma cell lines and primary cultures of human Kupffer cells have demonstrated that DENV is able to infect hepatocytes and Kupffer cells^19,26–28^. Several molecules that act either as receptors for the virus or as attachment factors that facilitate viral concentration on the membrane before binding to receptors have been identified in liver cells^29,30^. In humans, the viral genome could be amplified from autopsy samples, and DENV antigen has been detected in hepatocytes, endothelial cells and Kupffer cells^16,17,31^.

Histological changes, such as fatty changes (macro- and microvesicular steatosis), hepatocellular necrosis, hepatocyte swelling followed by ballooning degeneration, Kupffer cell hyperplasia and destruction, Councilman bodies and cellular infiltrates at the portal tract, hemorrhage foci and edema, were noted in necropsy samples of DEN fatal cases^14,32–39^. Most reports of histopathologic changes are based on samples obtained from fatal cases. Thus, it is difficult to assess the degree of changes present in patients with milder disease, which leads researchers to seek animal models to study DEN pathogenesis in different organs.

Genetic variability of DENV can be attributed to the lack of a mechanism underlying transcriptional fidelity. At each round of genome replication, a mutation is produced^40,41^. Phylogenetic and molecular epidemiological data characterize DENV into different genotypes, which are generally associated with different geographic areas^42^. Based on the complete sequencing of the E gene, Weaver and Vasilakis^43^ proposed the characterization of DENV-1 into 5 genotypes, DENV-2 into 6 genotypes, DENV-3 into 5 genotypes and DENV-4 into 4 genotypes.

In Brazil, the DENV-2 S*outheast Asian*/American genotype is currently circulating. After its introduction in the country in the 1990s, two DENV-2 outbreaks occurred in Rio de Janeiro (1998 and 2008)^44^. Phylogenetic studies have shown that the strains from both epidemics belonged to the Asian/American genotype; however, isolates from the 2008 outbreak grouped together and gave rise to a new distinct lineage (Lineage II) from the lineage that was initially introduced in the country (Lineage I)^45,46^.

During the 1998 epidemic, more than 700 thousand cases were reported, mostly affecting the portion of the population between 20 and 40 years old^44,47^. In addition to introducing a new epidemiological profile of the disease that presents increased severity in children ≤ 15 years old, the 2008 epidemic led to 806,036 cases countrywide. In Rio de Janeiro, approximately 322,000 DEN cases and 252 deaths were reported^47–51^. DENV-2 Lineage II was associated with higher viremia in patients with SD than in patients with CD^52^, however, the role played by different lineages of a genotype is not completely understood.

The establishment of animal models is of great importance due to the lack of an effective tetravalent vaccine and a specific treatment for DEN and the need to understand the pathophysiological mechanisms leading to the different manifestations of the disease^53–55^.

Although BALB/c mice may be less susceptible to DENV infection^56^, viral replication and dissemination have already been observed in this murine model. When infected by an epidemic DENV-2 strain, BALB/c produces viremia between the 2nd and 11th days after infection^57^, and when adapted strains are used, viremia peaks on the 6th day postinfection^58^. Moreover, DENV-infected BALB/c present clinical signs similar to those observed in humans, such as increased transaminase levels and thrombocytopenia. Infection by neuroadapted strains cause severe disease with anorexia, weight loss, anemia, limb paralysis, and shock and lead to death^19,57–59^.

Similar to DEN human cases, multiple organs in BALB/c mice are affected by DENV infection, and the viral genome and antigen have been detected in the spleen, liver, brain, heart, lung, kidney and saliva^60–64^. Furthermore, histopathological studies on the livers of infected BALB/c mice have shown inflammatory cell infiltrates, intracellular edema, sinusoid capillary collapse, hemorrhage, hepatocellular vacuolization, increased binucleate hepatocyte population and hepatocyte death^19,57–59,65^.

Different serotypes and DENV strains may present differences in tissue tropism^20,60^. As previously mentioned, one of the factors that may interfere with the DENV infection outcome is the virulence of viral strains^13^. Here, we aimed to assess the susceptibility of BALB/c mice to two distinct DENV-2 lineages and characterize the impact of these lineages in the murine model liver.

## Results

No mice died over the course of this study. All animals were euthanized at 24 hpi, 48 hpi, 72 hpi, 7 dpi or 14 dpi. Clinical signs, such as petechiae, tremors or diarrhea, as well as neurological signs, such as paralysis, were not observed.

### Body temperature

At 72 hpi, there was a decrease in the mean temperature of both infected and noninfected mice (means: negative control = − 0.223 °C, Lineage I = − 0.67 °C and Lineage II = − 0.355 °C). After 7 and 14 dpi, a small temperature increase was observed in noninfected mice (means: 0.715 °C and 0.307 °C, respectively). The same did not occur with infected mice, whose mean temperature showed small decreases at 7 and 14 dpi (means: Lineage I = − 0.626 °C and − 0.006 °C, respectively; Lineage II = − 0.024 °C and − 0.24 °C, respectively). The difference between body temperature variation of the noninfected group and the group infected with Lineage I was significant at 7 dpi (*p* < 0.05).

### Body weight variation

**Figure 1:**
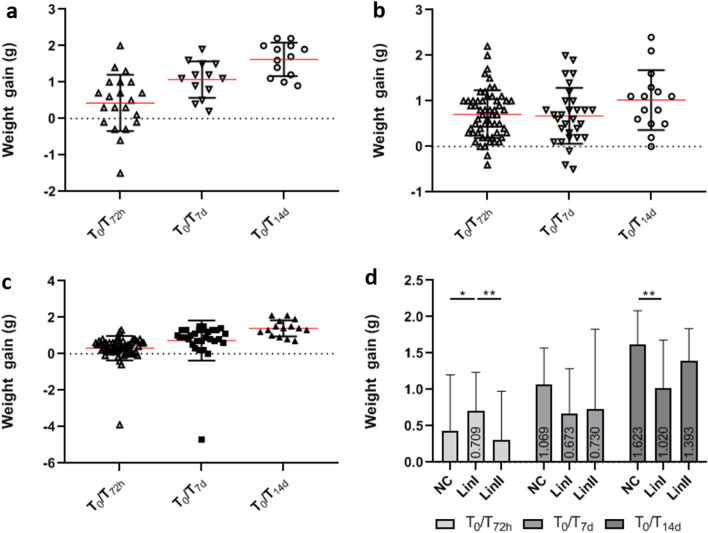
**Body weight gain of BALB/c mice uninfected and infected with DENV-2 Lineages before infection (T**_**0**_), **72 hpi (T**_**72h**_**), 7 dpi (T**_**7d**_**) and 14 dpi (T**_**14d**_**)**. (**a**) negative control; (**b**) DENV-2 Lineage I; (**c**) DENV-2 Lineage II; (d) comparison of body weight gain means. NC: (n: T_0_/ T_72h_ = 21; T_0_/ T_7d_ = 13; T_0_/ T_14d_ = 13); Lin I: (N: T_0_/ T_72h_ = 58; T_0_/ T_7d_ = 30; T_0_/ T_14d_ = 15); Lin II: (N: T_0_/ T_72h_ = 58; T_0_/ T_7d_ = 30; T_0_/ T_14d_ = 15). n: number of mice, NC: negative control, Lin: Lineage, hpi: hours post-infection, dpi: days post-infection, *p < 0.05, **p < 0.01.

On average, mice from all experimental groups gained weight; however, aside from Lineage I at 72 hpi (mean = 0.709 g), the weight gain of infected mice was lower compared to that of the control group. At 72 hpi, a small increase in body weight was observed in both noninfected and infected mice. The average weight gain of Lineage II-infected mice was 0.311 g, and the variation among noninfected mice was 0.429 g. On the seventh day after infection, noninfected mice and mice infected with Lineage II presented an increase in body weight (means: 1.069 g and 0.703 g, respectively), while the mean body weight of mice infected with Lineage I was slightly lower than that at 72 hpi (mean = 0.673 g). After 14 dpi, the mean for noninfected mice was 1.623 g, and for infected mice, it was 1.02 g (Lineage I) and 1.339 g (Lineage II). The difference between the body weight gain of the groups infected with Lineages I and II of DENV-2 was significant at 72 hpi (*p* < 0.01), and the difference between the negative control group and mice infected with Lineage I was significant at 7 dpi (*p* < 0.05) and 14 dpi (*p* < 0.001) (Fig. 1).

### Liver weight variation

**Figure 2:**
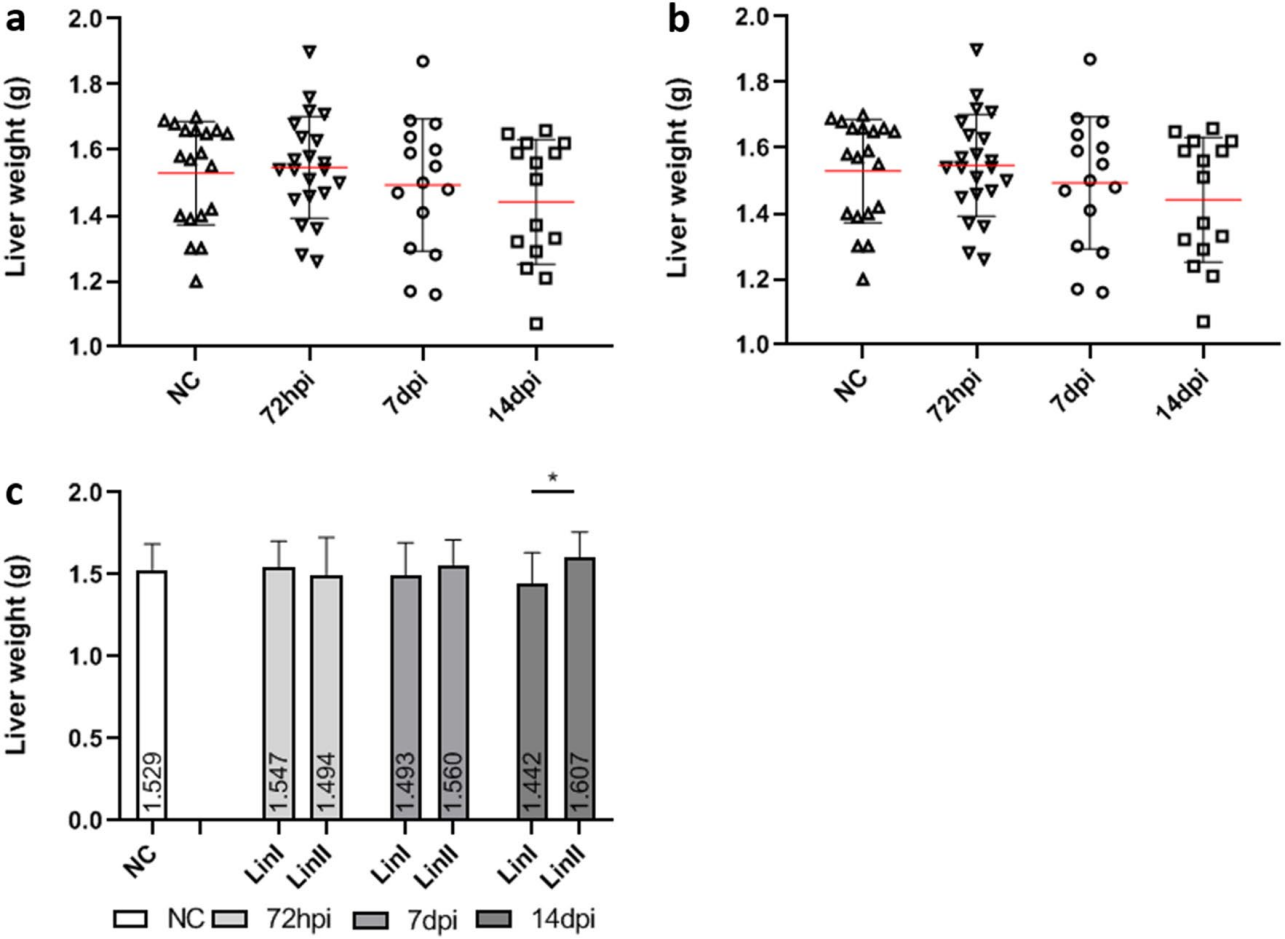
**Liver weight of BALB/c mice uninfected and infected with DENV-2 Lineages 72 hpi, 7 and 14 dpi.** (**a**) DENV-2 Lineage I; (**b**) DENV-2 Lineage II; (**c**) comparison of liver weight means. NC: (n = 19); Lin I: (n: 72 hpi = 22; 7dpi = 15; 14 dpi = 15); Lin II: (n: 72 hpi = 22; 7dpi = 15; 14 dpi = 15). n: number of mice, NC: negative control, Lin: Lineage, hpi: hours post-infection, dpi: days post-infection, *p < 0.05.

Comparing the mean liver weight of noninfected mice (1.529 g) and mice infected with Lineage I, it is possible to observe that the infected group presented heavier livers (mean = 1.547 g) at 72 hpi and the means were lower than that in control group after 7 dpi (1.493 g at 7 dpi and 1.442 g at 14 dpi). In mice infected with Lineage II, the opposite was observed. At 72 hpi, the mean liver weight (1.494 g) was lower than that of the control group and subsequently increased, presenting higher means of 1.560 g and 1.607 g at 7 and 14 dpi, respectively. The difference between the means of mice infected with Lineages I and II was significant at 14 dpi (p ≤ 0.05) (Fig. 2).

### Liver weight/ body weight ratio (%)

**Figure 3:**
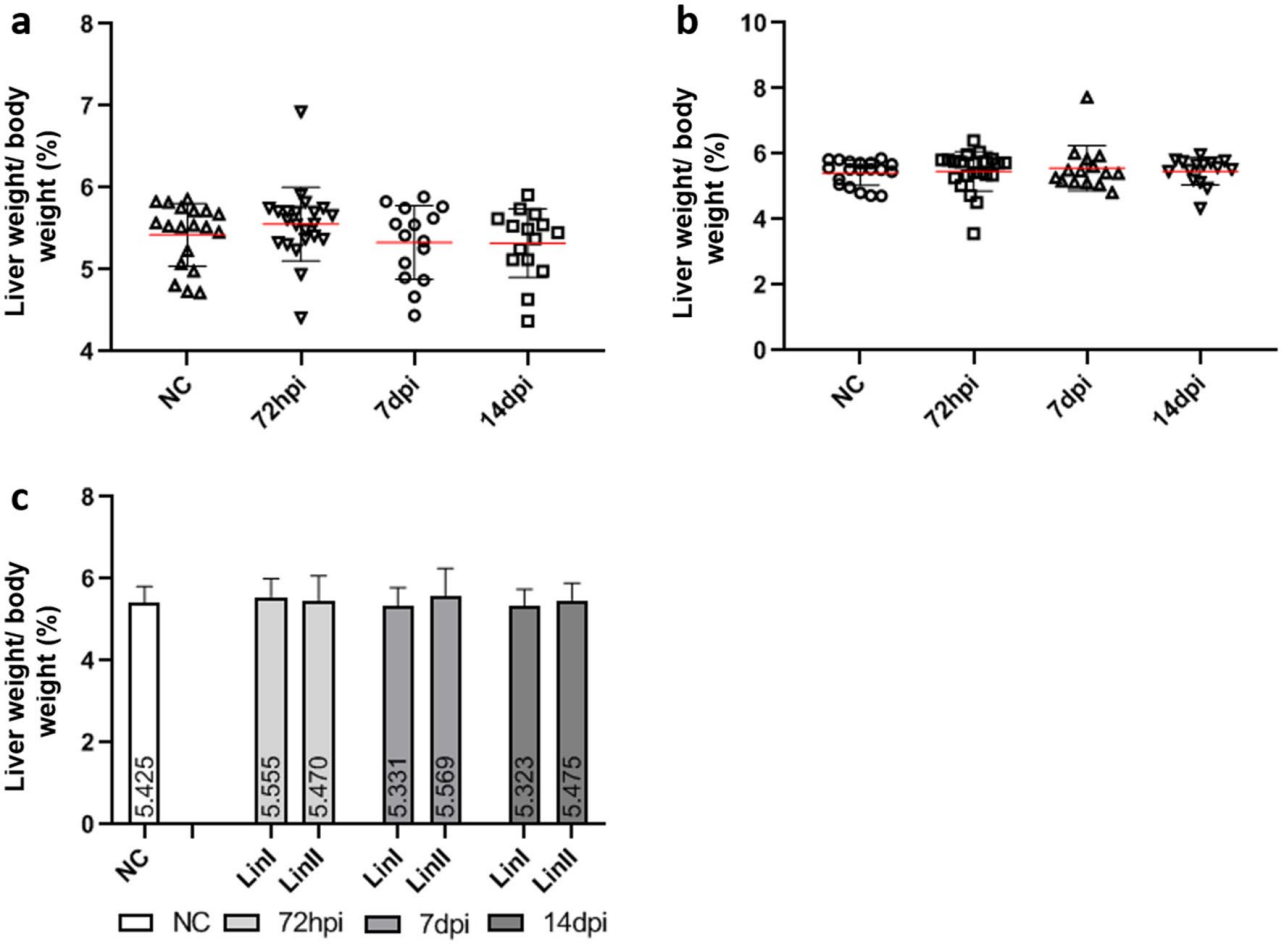
**Liver weight / body weight ratio (%) of BALB/c mice uninfected and infected with DENV-2 Lineages 72 hpi, 7 and 14 dpi.** (**a**) DENV-2 Lineage I; (**b**) DENV-2 Lineage II; (**c**) comparison of ratio means. NC: (n = 19); Lin I: (n: 72 hpi = 22; 7 dpi = 15; 14 dpi = 15); Lin II: (n: 72 hpi = 22; 7dpi = 15; 14 dpi = 15). n: number of mice, NC: negative control, Lin: Lineage, hpi: hours post-infection, dpi: days post-infection.

The average percentage of liver weight in relation to body weight of mice infected with Lineage I was slightly higher at 72 hpi (5.55%) than that observed in uninfected mice (5.42%). However, it decreased at subsequent kinetic points (7 dpi = 5.331%; 14 dpi = 5.323%). When comparing the averages of mice infected with Lineage II and uninfected mice, a slight increase was observed on the third and seventh days of infection (mean = 5.47% and 5.569%, respectively). After 14 days of infection, the liver weight/body weight ratio showed a slight decrease (mean = 5.75%); however, it remained greater than that of the control group (Fig. 3).

### Hematological parameters

#### Platelets

**Figure 4:**
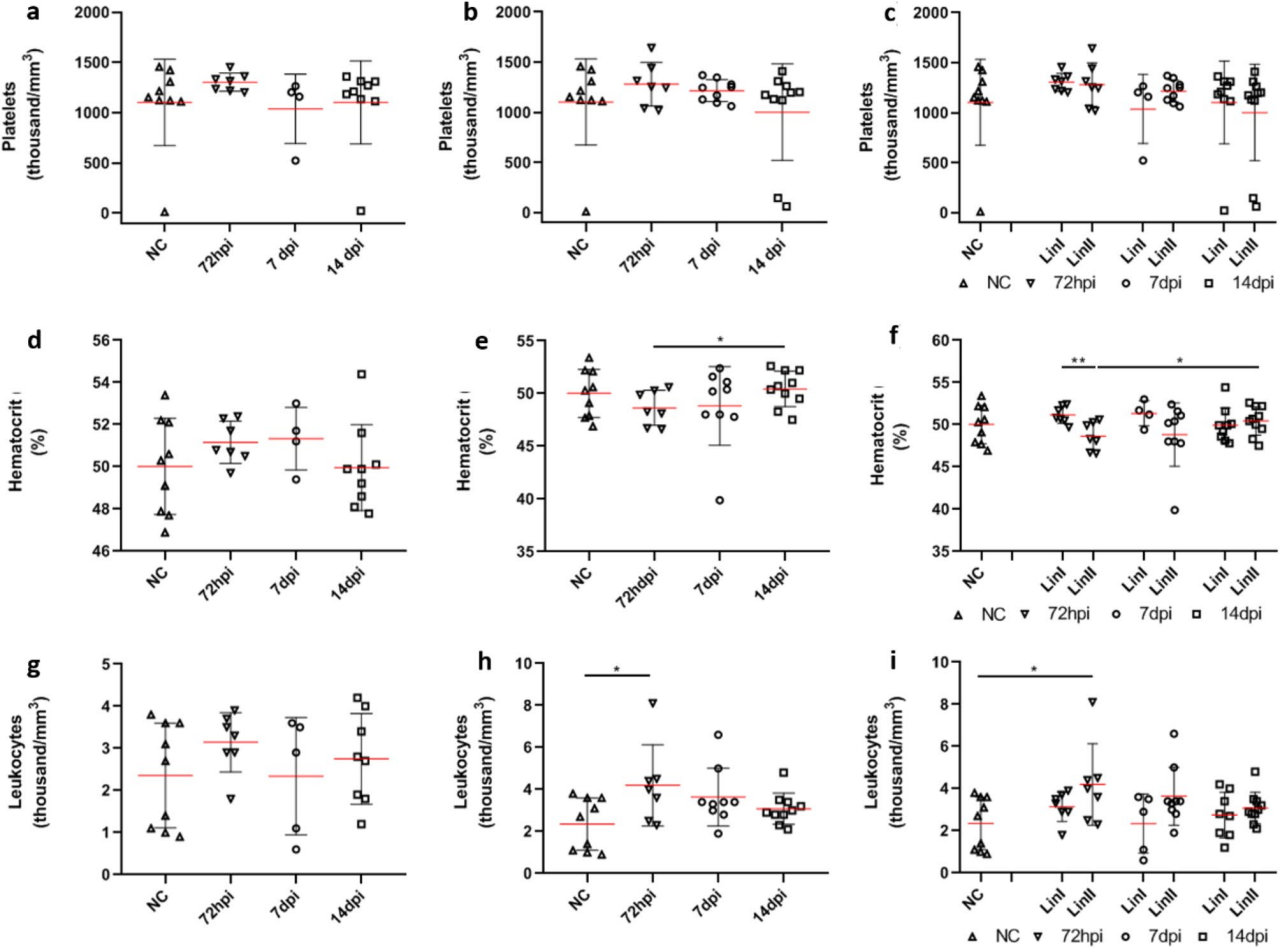
**Hematological parameters of BALB/c mice uninfected and infected with DENV-2 Lineages 72 hpi, 7 and 14 dpi.** Platelet count (thousands/mm^3^): (**a**) DENV-2 Lineage I; (**b**) DENV-2 Lineage II; (**c**) comparison of platelet count means. Hematocrit (%): (**d**) DENV-2 Lineage I; (**e**) DENV-2 Lineage II; (**f**) comparison of hematocrit means. Leukocyte count (thousand/mm^3^): (**g**) DENV-2 Lineage I; (**h**) DENV-2 Lineage II; (**i**) comparison of leukocyte count means. NC: (n = 10); Lin I: (n: 72 hpi = 10; 7dpi = 10; 14 dpi = 10); Lin II: (n: 72 hpi = 10; 7dpi = 10; 14 dpi = 10). NC: negative control, n: number of mice, hpi: hours post-infection, dpi: days post-infection, *p < 0.05, **p < 0.01.

The mean number of platelets present in blood samples from mice in the control group was 1103.89 thousand/mm^3^. In blood samples from mice infected with both DENV-2 strains, a slight increase was observed in the number of platelets at 72 hpi, with an average of 1304.43 thousand/mm^3^ for Lineage I and 1308.57 thousand/mm^3^ for Lineage II. On the seventh and fourteenth days after infection, the number of platelets in the infected samples decreased; however, except for the group infected with Lineage I at at 7 dpi and 14 dpi (means = 1038.7 thousand/mm^3^ and 1103.3 thousand/mm^3^, respectively) and the group infected with Lineage II at 14 dpi (mean = 1002.4 thousand/mm^3^), the infected group averages were not lower than that of the control group (Fig. 4).

#### Hematocrit

The HCT of blood samples from uninfected mice was 50.022% on average. In mice infected with DENV-2 Lineage I, the averages observed three and seven days after infection were higher at 51.157% and 51.325%, respectively. In mice infected with Lineage II, the HCT decreased at 72 hpi and 7 dpi (48. 643% and 48.822%, respectively) and increased on the fourteenth day (50.44%), slightly surpassing the mean of the negative control group (Fig. 4). The difference between the group infected with Lineage I and Lineage II was statistically significant (*p* < 0.01) at 72 hpi. The difference between mice infected with Lineage II at 72 hpi and 14 dpi was also significant (*p* < 0.05).

#### Leukocytes

Seventy-two hours after infection, the leukocyte count of blood samples from mice infected with DENV-2 Lineage I (mean = 3.143 thousand/mm^3^) and Lineage II (mean = 4.2 thousand/mm^3^) was higher than that observed in the control group (mean = 2.356 thousand/mm^3^). The difference between the control group and the group infected with Lineage II was statistically significant (*p* < 0.05). On the seventh day of infection, the white blood cell count of mice infected with Lineage I was lower than that of uninfected mice (2.34 thousand/mm^3^), and the average number of individuals infected with Lineage II was equal to 3.664 thousand/mm^3^. At 14 dpi, the group infected with Lineage I showed an average of 2.75 thousand/mm^3^, and the group infected with Lineage II showed an average of 3.08 thousand/mm^3^ (Fig. 4).

### Biochemical parameters

#### Aspartate aminotransferase (AST)

**Figure 5:**
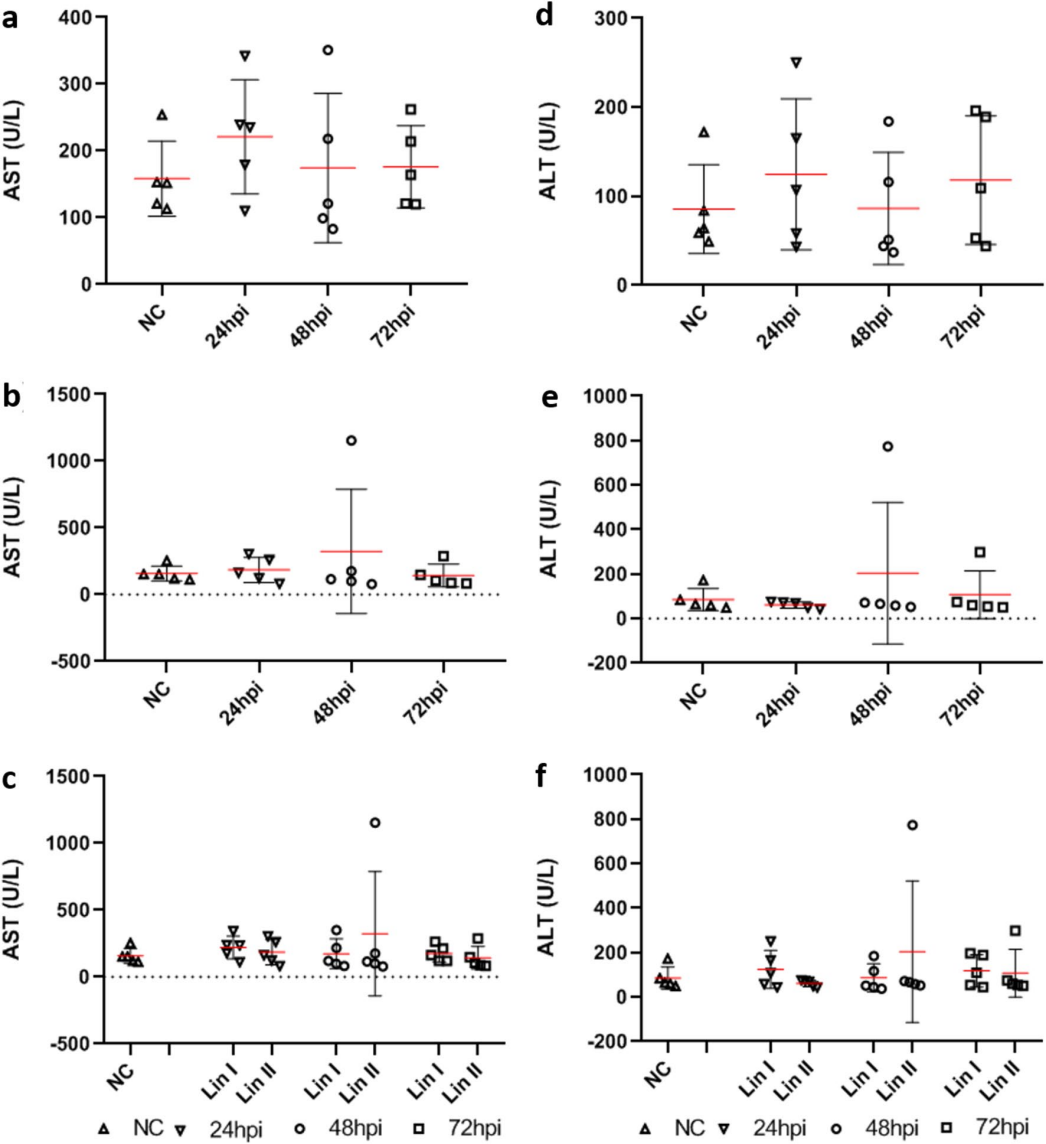
**Transaminases levels (U/L) of BALB/c mice uninfected and infected with DENV-2 Lineages 24, 48 and 72 hpi.** AST: (**a**) DENV-2 Lineage I; (**b**) DENV-2 Lineage II; (**c**) comparison of AST levels means. ALT: (**d**) DENV-2 Lineage I; (**e**) DENV-2 Lineage II; (**f**) comparison of ALT levels means. NC: (n = 5); Lin I: (n: 24 hpi = 5; 48 hpi = 5; 72 hpi = 5); Lin II: (n: 24 hpi = 5; 48 hpi = 5; 72 hpi = 5). NC: negative control, n: number of mice, hpi: hours post-infection.

The average level of AST in serum samples from uninfected mice was 158.6 U/L. Except for the group infected with Lineage II euthanized on the third day of infection (mean = 143.2 U/L), the infected mice generally showed increased levels of the enzyme when compared to the control group. In mice infected with Lineage I, the level of AST was higher on 24 hpi (mean = 221 U/L), decreased on the second day of infection (mean = 174 U/L) and then slightly increased at 72 hpi (mean = 176.2 U/L). In mice infected with Lineage II, the AST levels were also higher compared with the control group on the first day of infection (mean = 185.6 U/L); however, the concentration of the enzyme peaked at 48 hpi (mean = 324.8 U/L). On the third day after infection, AST levels were lower than those in the control group (Fig. 5).

#### Alanine aminotransferase (ALT)

The mean level of ALT present in the sera of uninfected mice was 85.6 U/L. In mice infected with Lineage I, the concentration of aminotransferase was higher in the serum of mice 24 hpi (mean = 144.6 U/L). On the second day of infection, the average was 86.4 U/L, and on the third day of infection, it was 118.2 U/L. In samples from mice infected with Lineage II at 24 hpi, the ALT levels were lower than those in the control group (mean = 61 U/L). The enzyme concentration reached its peak on the second day of infection (mean = 204 U/L) and decreased on the third day (mean = 107 U/L) (Fig. 5).

### Viral genome detection

Viral RNA was detected in two liver samples. One mouse was infected with Lineage I (1.2 × 10^–1^ copies of RNA/µl), and one mouse was infected with Lineage II (3.93 × 10^8^ copies of RNA/µl).

### Histopathology

**Figure 6:**
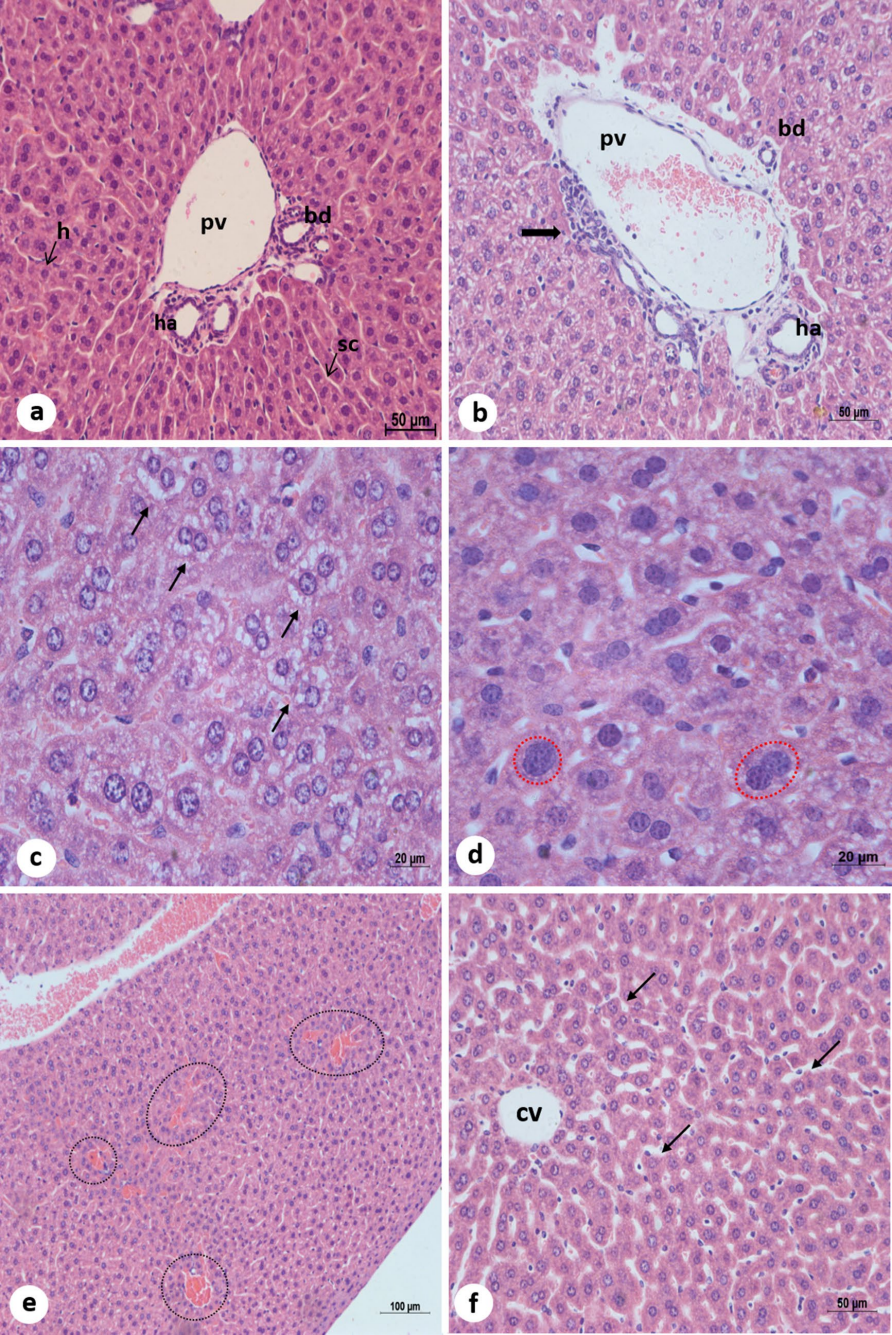
**Liver micrographs of BALB/c mice. H&E staining.** (**a**) negative control. (**b**–**d**) mice infected with DENV-2 Lineage I (Lin I) or II (Lin II). (**b**) (LinII) inflammatory infiltrate (arrow). (**c**) (Lin I) hepatic cell ballooning (arrows) (**d**) (Lin I) enlarged cell nucleus (red-dotted outline). (**e**) (Lin II) vascular congestion (dashed outline). (**f**) (Lin II) dilation of sinusoid capillaries (arrows). pv: portal vein. bd: bile duct. ha: hepatic artery. sc: sinusoid capillary. h: hepatocytes.

**Figure 7:**
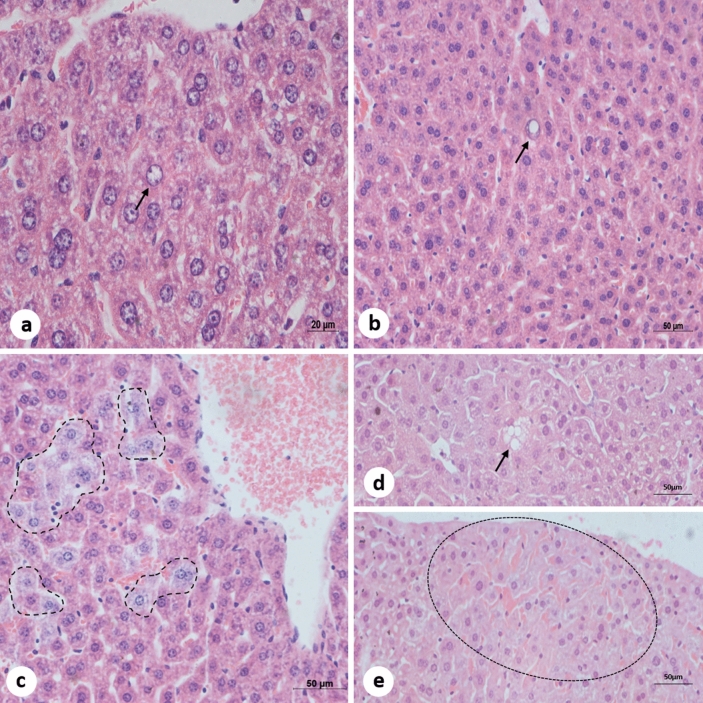
**Liver micrographs of BALB/c mice infected with DENV-2 Lineage I (Lin I) or II (Lin II). H&E staining.** (**a**) (Lin I), (**b**) (LinII) nuclear atypias (arrows). (**c**) (Lin II) signs of necrosis (dashed outline). (**d**) (Lin II) macrovesicular steatosis (arrow). (**e**) (Lin II) focal haemorrhage (dashed outline).

The images of histological sections (Figs. 6 and 7)are representative of changes observed in the liver samples of BALB/c mice infected with DENV-2 Lineage I or II and euthanized at 72 hpi. Table 1 shows the number of infected mice whose livers presented each alteration.

**Table 1:** Histopathological alterations observed in liver samples of BALB/c infected with DENV-2 Lineages I or II.

Alterations	DENV-2		
	Lineage I (%)	Lineage II (%)	Total (%)
Inflammatory cell infiltration	7/10 (70)	8/10 (80)	15/20 (75)
Hepatic cell ballooning	7/10 (70)	8/10 (80)	15/20 (75)
Enlarged cell nucleus	7/10 (70)	8/10 (80)	15/20 (75)
Vascular congestion	6/10 (60)	8/10 (80)	14/20 (70)
Sinusoid capillary dilation	9/10 (90)	1/10 (10)	10/20 (50)
Nuclear atypia	7/10 (70)	3/10 (30)	10/20 (50)
Necrosis	0/10 (0)	2/10 (20)	2/20 (10)
Macrovesicular steatosis	0/10 (0)	1/10 (10)	1/20 (5)
Haemorrhage	0/10 (0)	1/10 (10)	1/20 (5)
Signs of necrosis	0/10 (0)	1/10 (10)	1/20 (5)

Number of mice whose livers presented the alteration/total number infected mice.

Liver samples from noninfected mice showed intact parenchyma, with no signs of edema, congestion, steatosis or hemorrhage. The hepatocytes did not show nuclear or cytoplasmic alterations. Sinusoid capillaries were not dilated and did not present cellular infiltration or hemorrhaging, and Kupffer cells did not present histopathological alterations (Fig. 6a). Morphological alterations induced by both DENV-2 lineages in BALB/c mouse livers were focal and similar except for two cases. The most frequently observed alterations were inflammatory infiltrates, which is commonly observed next to the portal area (Fig. 6b), hepatocyte swelling and cytoplasmic loss, suggesting hepatic cell ballooning (Fig. 6c), hepatocyte nuclear area enlargement and chromatin pattern alterations (Fig. 6d), and vascular congestion (Fig. 6e). Sinusoid capillary dilation (Fig. 6f), which is more pronounced around centrolobular veins, and nuclear atypia, which is characterized by nuclear inclusions (Fig. 7b) or thin and regularly scattered chromatin, giving a relatively homogeneous appearance to the nuclei (Fig. 7a), were also seen. Only one mouse infected with DENV-2 Lineage II presented lipid droplets within the liver parenchyma (Fig. 7d), suggesting macrovesicular steatosis and focal hemorrhage (Fig. 7e). Signs of necrosis, or citoplasmic rarefaction, were only seen in samples infected with Lineage II (Fig. 7c).

### Morphometry

**Figure 8:**
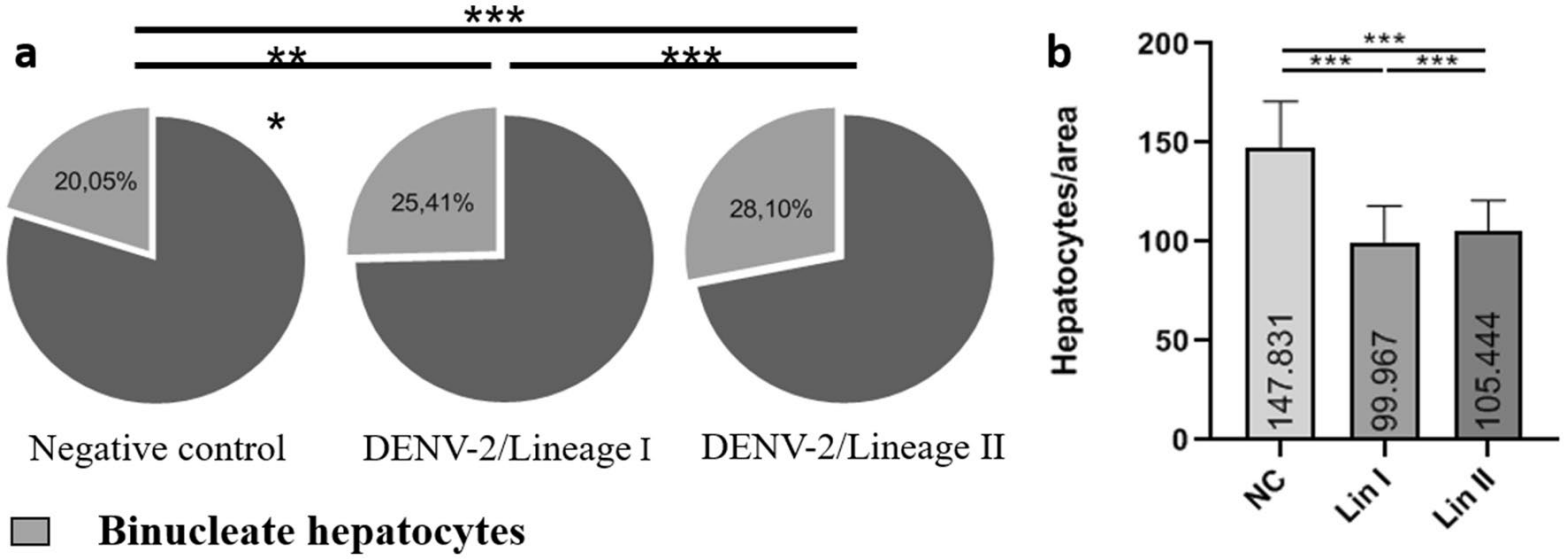
**Hepatocyte count from samples of BALB/c mice uninfected and infected with DENV-2 Lineages I or II euthanized 72 hpi**. (**a**) population of binucleated hepatocytes in negative control samples; (**b**) population of binucleate hepatocytes in samples infected with DENV-2 Lineage I; (**c**) population of binucleate hepatocytes in samples infected with DENV-2 Lineage II; (**d**) hepatocyte count per captured area. NC: negative control; Lin: Lineage, hpi: hous post-infection. ***p < 0.001.

Hepatocyte counting showed a significant increase in the number of binucleate cells in mice infected with both DENV-2 lineages at 72 hpi compared with the control group (Fig. 8a). Morphometrical analysis showed that the percentage of binucleate hepatocytes was 25.41% in mice infected with Lineage I (*p* < 0.001), 28.1% in mice infected with Lineage II (*p* < 0.001) and 20.05% in noninfected mice. However, when all hepatocytes were accounted for, a significant decrease in the cell population of infected mice was noted. Samples of mice infected with Lineage I presented 32.4% fewer cells than control samples (*p* < 0.001), and samples of mice infected with Lineage II presented 28.7% fewer cells (*p* < 0.001) (Fig. 8b).

## Discussion

The DENV-2 introduction in Rio de Janeiro in 1990 led to the first cases of SD in the state and country^44,66^. Its re-emergence in 2007 caused a great DEN epidemic in 2008^49^. Although the DENV-2 that reemerged in 2007–2008 still belongs to the Southeast Asia genotype, two distinct strains (Lineage I and Lineage II) within this genotype have been described^45,46^. Genetic variations between both strains were observed; however, no consistent differences in the genome that could be correlated with the severity of the disease have been identified to date^46,67^. Therefore, the factors that determine why certain DEN patients present mild symptoms and others develop severe disease are still not well defined. This study assessed the susceptibility of BALB/c mice to two distinct strains of DENV-2 and characterized alterations induced by each strain.

DEN patients may manifest acute fever that persists for three to seven days after the virus incubation period^68^. In this study, the average temperature variation was very small. Interestingly, only the control group showed a slight increase in temperature at 7 and 14 dpi. In studies carried out in BALB/c mice infected with DENV-3 and DENV-4 by our group, an increase in temperature at 72 hpi was observed^64,69^ (unpublished data). However, the absence of a temperature rise and other clinical signs in this same animal model infected with DENV-2 have been previously reported^19,65^ and may be related to asymptomatic DEN because only 25% of the cases of the disease show symptoms^12^.

Although no neurological signs were observed in this study, paralysis was reported in BALB/c mice infected with DENV-2^58,65^. In addition, DENV-1-infected BALB/c mice experienced mild hemorrhage in the brain but no signs of neurological disease^60^.

The weight gain among infected mice was lower than that of the control group, which may be associated with a loss of appetite, as observed in human DEN cases and immunocompetent models infected with DENV-2^11,58,70–72^. A cohort study conducted with DEN patients in a geriatric clinic reported that anorexia was the main complaint and that weight loss was greater during infection^72^. Among children, anorexia was reported in 78% of DF cases and in 91.2% of patients presenting DHF^70^. In DENV-3-infected mice, Caldas^64^ observed a slight increase in mouse weight in comparison to the control group; nevertheless, the actual weight variation of each individual was not assessed (unpublished data).

During DENV infection, changes in blood component counts are commonly observed and indicate disease prognosis. Among the changes, thrombocytopenia (platelet count below 100,000/mm^3^) is a hallmark for both mild and severe forms of DEN and may result from infection of bone marrow hematopoietic cell populations, which reduces their proliferative capacity^73,74^, platelet deposition in the microvascular bed, aggregation with leukocytes or destruction from peripheral blood^75^. At 72 hpi, the platelet count of infected mice was higher than that of the control group. At subsequent times of infection, the platelet count decreased and reached values slightly lower than those of the control group. A decrease in platelet count is to be expected and has been observed in immunocompetent mice infected with DENV-2^58^; however, the slight thrombocytosis at 72 hpi, which was also observed in mice infected with DENV-3^64^ (unpublished data), could be a response to factors triggered by infection that increase thrombopoietin production^76^.

Hemoconcentration can be observed as a result of plasma leakage^11^. An increase of the HCT of 20% over the baseline is a sign of DHF, and its maximum elevation coincides with shock^77^. HCT significantly increased in BALB/c mice infected with DENV-3^64^ (unpublished data). Mice infected with DENV-2 Lineage I presented a very slight increase (less than 2%) in HCT at 72 hpi and 7 dpi, and this observation was corroborated by histopathological analyses since plasma leakage was not observed.

The leukocyte count is variable, and although leukopenia is more frequently reported, there are cases of leukocytosis associated with SD^78^. Our study showed an increase in the leukocyte count of mice infected with both lineages of DENV-2; moreover, this change was more preeminent in mice infected with Lineage II. After peaking at 72 hpi, the leukocyte count started decreasing. Another mouse model infected with DENV-2 also showed leukocytosis followed by leukopenia^79^, and DENV-3-infected BALB/c mice presented a decrease in the number of leukocytes at 72 hpi, 7 dpi and 14 dpi^64^.

Liver involvement is commonly seen in DEN, and although more frequently associated with SD, it is also present in nonsevere cases of DEN^18,80^. Liver function abnormalities induced by DENV infection range from a mild rise in transaminase and bilirubin levels to acute liver failure, which may lead to death^22,81,82^. ALT and AST are considered indicators of liver abnormalities, as they are released into the bloodstream following liver cell injury^83^. Elevated levels of these enzymes are an early marker of DEN. Transaminase levels are higher in patients presenting DHF or DSS, and the increase is usually mild or moderate; however, an increase in enzyme levels of more than tenfold has been reported^20,22,84^. An increase in transaminase levels has also been seen in immunocompetent mice infected with DENV-2^19,57,59^. Our studies also showed an increase in AST and ALT levels at all times of infection in mice infected with Lineage I, while samples infected with Lineage II showed higher values at 24 and 48 hpi (AST) and 48 and 72 hpi (ALT).

The average liver weight of mice infected with both DENV-2 strains increased slightly and exceeded that of the control group at 72 hpi in mice infected with Lineage I and at 7 and 14 dpi in mice infected with Lineage II. Liver enlargement is commonly observed in DEN, mainly in SD cases^11,85^, and may result from edema due to vascular permeability or accumulation of fat within hepatocytes^2,86^. A study carried out with SD patients showed a high prevalence (72.7%) of hepatomegaly and associated painful hepatomegaly with increased levels of ALT^85,87^. When analyzing the liver weight/body weight ratio, we observed that it varied similarly to the liver weight, with this ratio higher at 72 hpi on average in mice infected with Lineage I and at all points of infection in the Lineage II group. These results suggest that the heavier liver in infected mice is not solely related to body weight gain and may be a consequence of infection. The reduction in body weight gain observed in infected mice and detection of the viral genome in macerates of the liver reinforce this hypothesis.

DENV infection-induced histopathological changes can be observed in the livers of experimentally infected immunocompetent mice as well as in autopsy samples^14,19,34,57,58,65,88,89^. Although only two liver samples tested positive for the viral genome (Lineage I: 1/10 and Lineage II: 1/10), a number of alterations were observed in our samples. The micrographs presented are representative of alterations observed in our samples. Alterations were focal, as reported elsewhere^88,90^. The most frequently observed change was inflammatory cell infiltration, which corroborates previous findings in BALB/c mice infected with DENV-2, -3 and -4^57,58,64,69,88^. Upon DENV infection, Kupffer cells and macrophages release cytokines and chemokines that activate inflammatory cells. Vasodilation is a result of proinflammatory cytokine release by Th1 cells^20^. Sinusoid capillary dilation was more frequently observed in samples infected with Lineage I (90%), whereas the frequency among samples infected with the other lineage was 10%. This alteration has been observed in other studies using the same mouse model^64,88,90^.

Hepatic cell ballooning is presumably caused by the influx of fluids into the cell due to damage to the cytoplasmic membrane^91^. This alteration was present in 70% and 80% of samples infected with Lineage I and Lineage II, respectively, and has been reported as a result of viral infection^92,93^. DENV infection is known to alter lipid metabolism^94^. Macrovesicular steatosis was observed in one sample infected with Lineage II. These changes were observed in studies performed by our group with BALB/c mice infected with DENV-3 and -4^64,69^ (unpublished data). Intracellular accumulation of fat occurs in different DENV-infected human cells as well as in the *A. albopictus* C6/36 cell line and may play a role in viral production^95,96^.

In this study, enlargement of the nucleus was observed. Kariocytomegaly can be caused by hepatocyte polyploidy. There is no mention of hepatocyte kariocytomegaly in DEN; however, the increased number of hepatic cells with enlarged nuclei has been associated with liver damage^97,98^. Other atypical-looking nuclei presented inclusions and altered chromatin patterns, which were also seen in the livers of BALB/c mice infected with DENV-3^64^ (unpublished data). Nuclear inclusions accumulate, in the nuclear matrix, substances that are not found in the nucleus under normal circumstances and can be caused by viral infection. Hepatocytes may be a result of glycogen accumulation^99^. Paes^57^ has also observed nuclear inclusions in liver samples of BALB/c mice and described them as lipid-like. Further investigation is necessary to identify the nature of the inclusion.

Hepatocyte polyploidization and binucleation are features of liver growth and physiology, can be associated with disease and could be favorable for pathogens or induced by them to improve survival^100^. Moreover, binucleate cells may be more capable of responding to a major demand for protein synthesis^101–103^. Our infected samples presented a significant increase in binucleation. The same change was observed in BALB/c mice by Sakinah^65^ and by our group in an ongoing study on reinfection^104^. Binucleate cells may be formed by nuclear division or cell fusion^105,106^, and several viruses have fusogenic activity^107,108^. Grizzi^100^ suggested that binucleation is a cell’s response to hepatic illness because it increases with progression of the necroinflammatory state. It can also be a sign of tissue regeneration because it was reported to increase after partial hepatectomy^109^. Binucleation may have increased as a response to infection. A decrease in the total number of hepatocytes was observed in infected mice, which may indicate cell death. Indeed, some of our samples presented signs of necrosis, as seen in other studies with DENV-2^57,58,88^ and DENV-4^69^ (unpublished data).

SD disease is associated with extensive involvement of the endothelium^74^. Vascular permeability plays an important role in SD pathogenesis^11,110^. Studies have shown that an increase in the number of infected hepatic endothelial cells coincides with the onset of SD^111^ and that vascular permeability is caused by inflammatory mediators rather than by infection of endothelial cells or cell death^110,112,113^. Although ours is not a SD model, focal hemorrhage was observed in one liver sample, suggesting altered vascular permeability. This finding is in accordance with human case reports^14,34^ and studies of this same experimental model infected with DENV-3 and DENV-4^64,69^ (unpublished data).

The outcome of infection and tissue tropism can be influenced by infective strain virulence^13,20,60^. Indeed, a study carried out with patient sera reported that the viremia level of Lineage II samples was two orders of magnitude higher than that of Lineage I samples and an increase in the number of SD cases occurred after DENV-2 strain emergence in 2007^52^. Although the viral genome was detected in only two samples (one of each lineage), the titer found in the liver of mice infected with Lineage II was higher.

In this study, two groups of BALB/c mice were experimentally infected with either Lineage I or Lineage II. No rise in temperature was observed among infected mice. A decrease in body weight gain was observed in infected mice. Although the 72 hpi mean weight gain of mice infected with Lineage II was significantly lower than that of mice infected with the other lineages (*p* < 0.01), at the end point of the experiment (14 dpi), it was inferior among mice infected with Lineage I (*p* < 0.01). It is noteworthy that while infected mice gained less weight, the livers of infected individuals were heavier than control group 72 hpi in Lineage I infected mice and 7 and 14 dpi in mice infected with Lineage II. The difference between the infected groups was statistically significant at 14 dpi (*p* < 0.05). At 72 hpi, the viral load of a liver sample infected with Lineage II was 3.93 × 10^8^ copies of RNA/µl (higher than Lineage I) and liver weight of this group was inferior compared to the other lineage, and these characteristics may be related to differences between the strains, the time of infection at which each lineage manifests symptoms, or host genetic factors. Nevertheless, more studies are necessary to generate conclusive findings.

The platelet count increased on the first three days of infection in both groups and declined subsequently. The mean number of platelets was lower than that in the control group at 7 dpi in mice infected with Lineage I, whereas the mean was inferior to that in the control group at 14 dpi in mice infected with Lineage II. Nonetheless, there was no statistically significant difference among the groups.

Similarly, the number of leukocytes increased at 72 hpi in both groups and decreased afterwards. The leukocyte count of Lineage II was higher than that of the control group at all times of infection, and at 72 hpi, the difference between means was statistically significant (*p* < 0.005). At seven dpi, the leukocyte count was slightly lower in mice infected with Lineage I than in the control group, but there was no significant difference between the infected and noninfected groups.

Regarding the HCT, the curves of the infected group presented opposite profiles. Although the HCT increased at 72 hpi and 7 dpi in mice infected with Lineage I, the values were lower than that of the control group 14 dpi; however, the HCT values in mice infected with Lineage II were greater than that of the control group at 14 dpi. Significant differences were observed in the HCT between the infected groups at 72 hpi (*p* < 0,001). Regarding mice infected with Lineage II, the difference was significant between the groups euthanized at 72 hpi and 14 dpi (*p* < 0.005).

The AST and ALT levels of the Lineage I group were higher than that in the control group at all times of infection, while the AST and ALT levels of the Lineage II group were lower than that in the noninfected mice at 24 hpi and 48 hpi, respectively. The levels of AST and ALT peaked at 24 hpi in mice infected with DENV-2 Lineage I (rise of 28.2% and 40.8% in relation to the control group, respectively) and at 48 hpi in mice infected with Lineage II (increase of 51.1% and 58% compared to the control group, respectively). The highest difference between the control group and Lineage II group concerning transaminase peak levels could be associated with the higher viral load detected in the sample infected with this lineage. Paes^57,59^ reported that AST and ALT peaked at the same time as the viral load in liver of BALB/c mice infected with DENV-2, although a cohort study conducted with both SD and nonsevere dengue patients did not find a correlation between viral titer and level of liver transaminases^18^. No significant difference was observed.

Histopathological alterations induced by the two lineages were focal and mostly similar, and the frequency at which alterations were observed varied. Some changes were seen at a much higher frequency in mice infected with Lineage I (sinusoid capillary dilation and nuclear atypia), while others were only seen in samples infected with the other lineage (macrovesicular steatosis and hemorrhage). There was a significant difference between the infected groups concerning the number of binucleate hepatocytes, which were higher in Lineage II-infected samples, and hepatocyte count, which was higher in Lineage I-infected samples. Morphological changes were mild compared to those reported in fatal cases^14,22,34^, and the samples were from mice euthanized at 72 hpi. However, in mice infected with Lineage II, the livers were heavier later in the infection; thus, it would be interesting to investigate tissue from mice euthanized at 7 and 14 dpi as well.

In addition to the circulation of viral strains of Asian origin, the current hyperendemicity of DEN highlights the need to investigate the role of these viruses in the occurrence of severe and fatal cases. Furthermore, after the reemergence of serotype 2 in 2017 and the consequent change in the predominance of the circulating serotype from DENV-1 to DENV-2^114^, there was an explosion of DEN cases in Brazil in 2019, with 905,912 probable cases reported until August^115^. Thus, the establishment of experimental models for the study of this serotype is relevant. Here, we have demonstrated that although the changes induced by the infection of the two DENV-2 strains are similar in terms of certain alterations, differences are observed in the infection timeline and intensity.

## Material and methods

### Ethical statements

All procedures performed in this study were approved by the Animal Ethics Committee (protocol L-041/2015) and the Human Research Ethics Committee (protocol 247/05) of Instituto Oswaldo Cruz, Fundação Oswaldo Cruz. All methods were carried out in accordance with the relevant guidelines and regulations and animal experimentation was in compliance with the ARRIVE guidelines for *in-vivo* studies.

### Virus

DENV-2 strains BR/RJ66985/2000 (GenBank #HQ012518) and BR/RJ0337/2008 (GenBank #HQ01253), representative of Lineage I and Lineage II^45,46^ were isolated from patient sera at Flavivirus Laboratory, IOC, FIOCRUZ, during the epidemics of 2000 and 2008, respectively. The serotype was confirmed by indirect immunofluorescence using a DENV-type-specific monoclonal antibody (3H5) and reverse transcription polymerase chain reaction (RT-PCR)^116,117^.

### Viral stock

Viral stocks were prepared by inoculating 100 µl of each strain into 175 cm^2^ cell culture bottles containing mosquito Ae. albopictus C6/36 cell line at a concentration of 5 × 10^5^ cells/ml. Titers of both strains (BR/RJ66985/2000:10^6^^66^ TCID_50_/1 ml and BR/RJ0337/2008:10^9^ TCID_50_/1 ml) were calculated by the Reed & Muench method^118^. The viruses did not undergo any passages through the mouse brain for neuroadaptation.

### Mice

For the experimental infection, two-month-old male BALB/c mice, provided by Instituto de Ciência e Tecnologia em Biomodelos (ICTB) of FIOCRUZ, were used. During the animal experimentation period, the animals were kept under controlled temperature, photoperiod, nutrition and hydration conditions.

### Study design

For infection with both lineages of DENV-2 (BR/RJ66985/2000 and BR/RJ0337/2008), BALB/c mice were inoculated by the intravenous route (IV) through the caudal vein. The inoculum volume was 100 µl, and the viral concentration was 10,000 TCID_50_/0.1 ml. The mice were anesthetized and euthanized (0.2 ml of ketamine, xylazina and tramadol solution) at 24, 48, 72 h postinfection (hpi) and 7 or 14 days postinfection (dpi) according to their experimental group. Blood was sampled by cardiac puncture before euthanasia and centrifuged in a refrigerated centrifuge (4 °C) for 10 min at 5000 rotations per minute to separate the serum from the cellular components. Liver samples destined for morphological analysis were fixed in Millonig buffered formalin. The samples destined for molecular studies were stored in a -80 °C freezer. Biochemical and hematological tests were carried out immediately after sample collection. Body temperature and weight were verified preinfection and before euthanasia and at 72 hpi, 7 dpi and 14 dpi. All liver samples were weighed immediately after harvesting, and to evaluate whether the liver weight increase was due to DENV infection and not just a consequence of body weight gain, a ratio (100 × liver weight/body weight) between both measurements was calculated. Noninfected mice were used as negative controls.

### Body temperature

**Figure 9:**
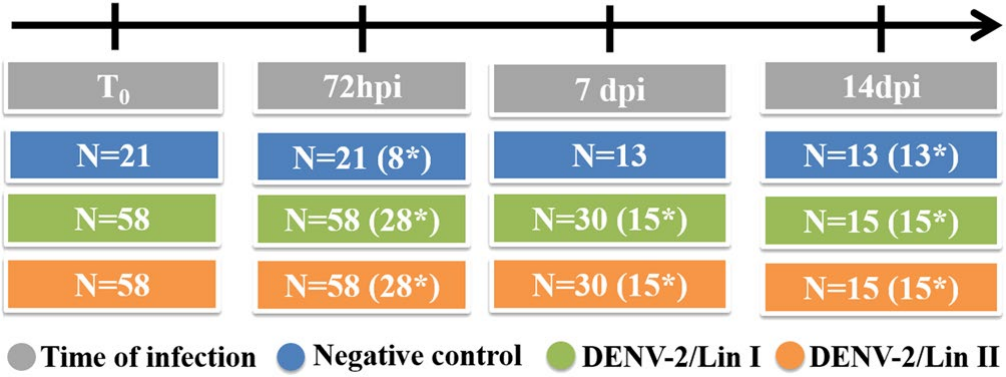
**Number of mice used in clinical analysis at each time of infection.** T_0_: before infection, hpi: hours post infection, dpi: days post infection, (*) mice euthanized at that time of infection, Lin: Lineage.

The body temperature of noninfected (n = 21) and infected (n = 58/lineage) mice was verified immediately before infection (T_0_) and at 72 hpi, 7 dpi and 14 dpi, and variation means (72 hpi-T_0_, 7 dpi-T_0_ and 14 dpi-T_0_) were calculated (Fig. 9, Table 2). Temperature was determined by dipping the measuring extremity of a digital thermometer in mineral oil and then gently inserting it into the mouse rectum for one minute.

**Table 2:** Number of mice used for clinical analysis and liver weight analysis.

	Body temperature/weight				Liver weight		
	T_0_	72hpi	7dpi	14dpi	72hpi	7dpi	14dpi
DENV-2/Lin I	58	58	30	15	22	15	15
DENV-2/Lin II	58	58	30	15	22	15	15
Negative Control	21	21	13	13			19
Total				137			123

T_0_: before infection, hpi: hours post infection, dpi: days post infection, Lin: Lineage.

### Body weight variation

The body weights of noninfected (n = 21) and infected (n = 58/lineage) mice were measured immediately before infection (T_0_) and at 72 hpi, 7 dpi and 14 dpi, and the variation (72 hpi-T_0_, 7 dpi-T_0_ and 14 dpi-T_0_) in the mean weight was calculated (Fig. 9, Table 2).

### Liver weight

Livers of noninfected (n = 19) and infected (n = 52/lineage) mice were weighed immediately and at 72 hpi, 7 dpi and 14 dpi, and the mean weight was calculated (Table 2).

### Hematological analysis

For both DENV-2 lineages, 30 mice were infected. The mice were divided into three groups of 10 animals, and each group was euthanized at different times after infection (72 hpi, 7 dpi and 14 dpi). Before euthanasia, the mice were anesthetized and blood was collected by cardiac puncture. Blood from noninfected mice (n = 10) was collected on the same day as the 14 dpi group. To avoid coagulation, samples were stored in EDTA-coated tubes. Platelets and leucocytes were counted, and hematocrit (HCT) was measured in collaboration with ICTB utilizing a Poch 100-iV DIFF platform (Sysmex, Kobe) (Table 3).

**Table 3:** Number of mice used for histopathological, hematological and biochemical analysis and viral RNA quantification.

N = 130 mice	Histopathology/qRT-PCR	Hematological analysis			Biochemical analysis		
	72 hpi	72 hpi	7 dpi	14 dpi	24 hpi	48 hpi	72 hpi
DENV-2 Lin I	10	10	10	10	5	5	5
DENV-2 Lin II	10	10	10	10	5	5	5
Negative control	5	10	5				
Total	25			70			35

hpi: hours post infection, dpi: days post infection, Lin: Lineage.

### Biochemical analysis

For both DENV-2 lineages, 15 mice were infected. The mice were divided into three groups of five animals, and each group was euthanized at different times after infection (24, 48 and 72 hpi). Prior to euthanasia, the mice were anesthetized and blood was collected by cardiac puncture. Blood samples were then centrifuged for 10 min at 5000 rotations per minute to separate the serum from the cellular components. Blood from noninfected mice (n = 5) was collected on the same day as the 72 hpi group. ALT and AST blood levels were measured by dry chemistry testing using a Vitros 250 system (Ortho clinical—Jonhson & Jonhson) and in collaboration with ICTB (Table 3).

### Nucleic acid extraction and quantitative RT-PCR

For each DENV-2 lineage, 5 BALB/c mice were infected and euthanized at 72 hpi. Five noninfected mice were used as a negative control. Viral RNA was extracted from liver samples of infected and noninfected mice. Organ samples were macerated with 500 µl of Leibovitz medium (Sigma). RNA was extracted from 140 µl of supernatant of macerated liver samples by using a QIAmp Viral RNA mini kit (Qiagen) as described by the manufacturer’s protocol. Viral RNA quantitation was carried out using the primers and probes DEN2-R (5-ACCATAGGAACGACACATTTCC-3) and DEN2-F (5-CAACGCATTGTCATTGAAGGA-3) and (FAM-5-AGGGCCTTGATTTTCATCTTACTGACAGC-3-TAMRA). TaqMan Fast Virus One-step Master Mix (Applied Biosystems) was used for the amplification reaction. Five microliters of extracted RNA and a mix containing 12.5 µl of reaction mix, 1 µl of DEN2-F and DEN2-R primers (Sigma), 0.75 µl of DEN2-P probe, 3.65 µl of nuclease-free water (Gibco), 1 µl of MgSO_4_ and 0.5 µl of SuperScript III Platinum One-Step Quantitative RT-PCR (Invitrogen) were applied to a 96-well microplate. The assay was performed using a 7500 FAST platform (Applied Biosystems). Thermal cycling parameters were as follows: reverse transcription at 50 °C for 15 min (min), 1 cycle of enzyme activation at 95 °C for 2 min, 1 cycle of denaturation at 95 °C for 15 s, 40 cycles of annealing/elongation at 60 °C for 1 min (Table 3).

### Bright field microscopy

For each DENV-2 lineage, 10 mice were infected. Five noninfected mice were used as a negative control. At 72 hpi, the mice were euthanized and liver samples were collected and fixed in Millonig buffered formalin. The samples were then dehydrated in decreasing concentrations of ethanol, clarified in xylene and embedded in paraffin. Tissue Sects. (5 µm thick) were obtained using a microtome and stained with hematoxylin and eosin (H&E) for posterior analysis with a bright field microscope (AxioHome, Carl Zeiss). All procedures were performed in collaboration with Pathology Laboratory, IOC/FIOCRUZ (Table 3).

### Histomorphometry

The morphometric analysis goal was to quantify the number of binucleated hepatocytes as well as the total number of hepatocytes in each group of mice. Fifteen glass slides containing histological sections of liver of BALB/c mice euthanized at 72 hpi and stained with H&E (five from noninfected mice, five from mice infected with each lineage) were analyzed. Thirty images of random areas were captured at 40 × magnification using a digital camera coupled to a bright field microscope (AxioHome, Carl Zeiss). Cells from a total of 450 areas were counted. The values obtained were then compiled by group, and the mean was calculated.

### Statistical analysis

A database was constructed with the data collected during the experiment. T-tests were performed using SPSS 25 software (IBM) and graphics were constructed using GraphPad Prism 8.0.1 software. P values of p ≤ 0.05 were considered statistically significant.

## References

[1] World Health Organization (2013). Sustaining the drive to overcome the global impact of neglected tropical diseases: second WHO report on neglected tropical diseases.

[2] Guzman MG, Harris E (2015). Dengue. Lancet.

[3] Murray NE, Quam MB, Wilder-Smith A (2013). Epidemiology of dengue: past, present and future prospects. Clin. Epidemiol.

[4] Bhatt S (2013). The global distribution and burden of dengue. Nature.

[5] Stanaway JD (2016). The global burden of dengue: an analysis from the Global Burden of Disease Study 2013. Lancet Infect Dis.

[6] Gubler DJ (2002). Epidemic dengue/dengue hemorrhagic fever as a public health, social and economic problem in the 21st century. Trends Microbiol..

[7] Wengler G, Wengler G, Gross HJ (1978). Studies on virus-specific nucleic acids synthesized in vertebrate and mosquito cells infected with flaviviruses. Virology.

[8] Rice CM (1985). Nucleotide sequence of yellow fever virus: implications for flavivirus gene expression and evolution. Science.

[9] Lindenbach BD, Rice CM (2003). Molecular biology of flaviviruses. Adv. Virus Res..

[10] World Health Organization. [https://www.who.int/] Geneva: Dengue control. Epidemiology. Avaiable in : http://www.who.int/denguecontrol/epidemiology/en/. Accessed: 12 Aug 2020.

[11] Gubler DJ (1998). Dengue and dengue hemorrhagic fever. Clin. Microbiol. Rev..

[12] World Health Organization (2009). Dengue Guidelines for Diagnosis, Treatment, Prevention and Control –.

[13] Halstead S. B. Pathogenesis of Dengue: Dawn of a new era. *F1000Research* 4, *F1000 Faculty Rev-1353*. 10.12688/f1000research.7024.1. (2015).

[14] Póvoa TF (2014). The pathology of severe dengue in *multiple organs of human fatal cases: histopathology, ultrastructure and virus replication*. PLoS ONE.

[15] Samanta, J., & Sharma, V. Dengue and its effects on liver. *World J Clin Cases* 3(2), 125–131. 10.12998/wjcc.v3.i2.125. (2015). 10.12998/wjcc.v3.i2.125 PMC4317605 25685758

[16] de Araújo JM (2009). A retrospective survey of dengue virus infection in fatal cases from an epidemic in Brazil. J. Virol. Methods..

[17] Lima M (2011). A new approach to dengue fatal cases diagnosis: NS1 antigen capture in tissues. PLOS Negl. Trop. Dis..

[18] Fernando S (2016). Patterns and causes of liver involvement in acute dengue infection. BMC Infect. Dis..

[19] França RF, Zucoloto S, da Fonseca BA (2010). A BALB/c mouse model shows that liver involvement in dengue disease is immune-mediated. Exp. Mol. Pathol..

[20] Dissanayake HA, Seneviratne SL (2018). Liver involvement in dengue viral infections. Rev. Med. Virol..

[21] Trung DT (2010). Liver involvement associated with dengue infection in adults in Vietnam. Am. J. Trop. Med. Hyg..

[22] Kularatne S (2018). Heart and liver are infected in fatal cases of dengue: three PCR based case studies. BMC Infect. Dis..

[23] Devarbhavi H, Ganga D, Menon M, Kothari K, Singh R (2020). Dengue hepatitis with acute liver failure: clinical, biochemical, histopathological characteristics and predictors of outcome. J. Gastroenterol. Hepatol..

[24] Kuo CH (1992). Liver biochemical tests and dengue fever. Am. J. Trop. Med. Hyg..

[25] Wang XJ (2016). Evaluation of aminotransferase abnormality in dengue patients: a meta analysis. Acta Trop..

[26] Cabrera-Hernandez A, Thepparit C, Suksanpaisan L, Smith DR (2007). Dengue virus entry into liver (HepG2) cells is independent of hsp90 and hsp70. J. Med. Virol..

[27] El-Bacha T (2007). Mitochondrial and bioenergetic dysfunction in human hepatic cells infected with dengue 2 virus. Biochim. Biophys. Acta.

[28] Marianneau P (1999). Infection of primary cultures of human Kupffer cells by Dengue virus: no viral progeny synthesis, but cytokine production is evident. J. Virol..

[29] Lühn K (2007). Increased frequencies of CD4+ CD25(high) regulatory T cells in acute dengue infection. J. Exp. Med..

[30] Cruz-Oliveira C (2015). Receptors and routes of dengue virus entry into the host cells. FEMS Microbiol. Rev..

[31] Nunes P (2019). 30 years of fatal dengue cases in Brazil: a review. BMC Public Health.

[32] Burke T (1968). Dengue haemorrhagic fever: a pathological study. Trans. R. Soc. Trop. Med. Hyg..

[33] Bhamarapravati N (1989). Hemostatic defects in dengue hemorrhagic fever. Rev. Infect. Dis..

[34] Basílio-de-Oliveira CA (2005). Pathologic study of a fatal case of dengue-3 virus infection in Rio de Janeiro Brazil. Braz. J. Infect. Dis..

[35] Seneviratne SL, Malavige GN, de Silva HJ (2006). Pathogenesis of liver involvement during dengue viral infections. Trans. R. Soc. Trop. Med. Hyg..

[36] Malavige GN, Fernando S, Fernando DJ, Seneviratne SL (2004). Dengue viral infections. Postgrad. Med. J..

[37] Leong AS, Wong KT, Leong TY, Tan PH, Wannakrairot P (2007). The pathology of dengue hemorrhagic fever. Semin. Diagn. Pathol..

[38] Limonta D (2012). Dengue virus identification by transmission electron microscopy and molecular methods in fatal dengue hemorrhagic fever. Infection.

[39] Kularatne, S. A., Gawarammana, I. B., & Kumarasiri, P. R. Epidemiology, clinical features, laboratory investigations and early diagnosis of dengue fever in adults: a descriptive study in Sri Lanka. *Southeast Asian J. Trop. Med. Public Health* 36(3), 686–692 (2005). 16124439

[40] Steinhauer DA, Domingo E, Holland JJ (1992). Lack of evidence for proofreading mechanisms associated with an RNA virus polymerase. Gene.

[41] Drake JW, Charlesworth B, Charlesworth D, Crow JF (1998). Rates of spontaneous mutation. Genetic.

[42] Chen R, Vasilakis N (2011). Dengue - quo tu et quo vadis?. Viruses.

[43] Weaver SC, Vasilakis N (2009). Molecular evolution of dengue viruses: contributions of phylogenetics to understanding the history and epidemiology of the preeminent arboviral disease. Infect. Genet. Evol..

[44] Nogueira RM, de Araújo JM, Schatzmayr HG (2007). Dengue viruses in Brazil, 1986–2006. Rev. Panam Salud Publica.

[45] Oliveira MF (2010). Two lineages of dengue virus type 2 Brazil. Emerg Infect Dis..

[46] Faria NR (2013). Twenty years of DENV-2 activity in Brazil: molecular characterization and phylogeny of strains isolated from 1990 to 2010. PLOS Negl. Trop. Dis..

[47] Barreto, M.L., & Teixeira, M.G. Dengue no Brasil: situação epidemiológica e contribuições para uma agenda de pesquisa. *Estud. av.* 22(64), 53–72. 10.1590/S0103-40142008000300005. (2008)

[48] Secretaria de Vigilância Sanitária/Ministério da Saúde. [https://portalarquivos2.saude.gov.br]. Brasília (DF): Relatório de casos de dengue. 2008. Available in: http://www.saude.rj.gov/Docs/Acoes/dengue/Relatorio.htm. (accessed: 12 Aug 2020)

[49] Teixeira MG, Costa M, Barreto F, Barreto ML (2009). Dengue: twenty-five years since reemergence in Brazil. Cad Saude Publica.

[50] Gibson G (2013). From primary care to hospitalization: clinical warning signs of severe dengue fever in children and adolescents during an outbreak in Rio de Janeiro Brazil. Cad Saude Publica.

[51] Macedo GA (2013). Virological surveillance for early warning of dengue epidemics in the State of Rio de Janeiro, Brazil. Trans. R. Soc. Trop. Med. Hyg..

[52] Nunes PC (2016). Dengue severity associated with age and a new lineage of dengue virus-type 2 during an outbreak in Rio De Janeiro Brazil. J. Med. Virol..

[53] Torresi J, Ebert G, Pellegrini M (2017). Vaccines licensed and in clinical trials for the prevention of dengue. Hum. Vaccin Immunother..

[54] Wilder-Smith, A., Ooi, E. E., Horstick, O., & Wills, B. Dengue. *Lancet (London, England) 393*(10169), 350–363. 10.1016/S0140-6736(18)32560-1. (2019). 10.1016/S0140-6736(18)32560-1 30696575

[55] World Health Organization – Dengue and Severe Dengue. Avaiable in: https://www.who.int/health-topics/dengue-and-severe-dengue#tab=tab_3. Accessed: 01 Mar 2021.

[56] Zompi S, Harris E (2012). Animal models of dengue virus infection. Viruses.

[57] Paes MV (2005). Liver injury and viremia in mice infected with dengue-2 virus. Virology.

[58] Atrasheuskaya A, Petzelbauer P, Fredeking TM, Ignatyev G (2003). Anti-TNF antibody treatment reduces mortality in experimental dengue virus infection. FEMS Immunol. Med. Microbiol..

[59] Paes, M. V. *et al* Hepatic damage associated with dengue-2 virus replication in liver cells of BALB/c mice. *Laboratory investigation; a journal of technical methods and pathology*, *89*(10), 1140–1151. 10.1038/labinvest.2009.83. (2009). 10.1038/labinvest.2009.83 19721415

[60] Tuiskunen, A. *et al* Phenotypic characterization of patient dengue virus isolates in BALB/c mice differentiates dengue fever and dengue hemorrhagic fever from dengue shock syndrome.*Virol. J.*, *8*, 398. 10.1186/1743-422X-8-398. (2011). 10.1186/1743-422X-8-398 PMC3170302 21835036

[61] Jácome FC (2015). Heart compromise and detection of dengue virus-like particles in cardiac tissue of experimentally infected murine model. IJRSB..

[62] Rasinhas A (2018). First detection of dengue virus in the saliva of immunocompetent murine model. Mem. Inst. Oswaldo Cruz.

[63] Salomão, N. G., *et a.* BALB/c mice infected with DENV-2 strain 66985 by the intravenous route display injury in the central nervous system. *Sci Rep*, *8*(1), 9754. 10.1038/s41598-018-28137-y. (2018). 10.1038/s41598-018-28137-y PMC6021404 29950590

[64] Caldas GC. Modelo Murino Imunocompetente Para Estudo Da Infecção Pelo Vírus Dengue 3: Aspectos Morfológicos, Viremia E Tropismo. Rio de Janeiro. Master’s thesis [Post-Graduation in Parasite Biology] – Instituto Oswaldo Cruz – Fiocruz; 2019.

[65] Sakinah S (2017). SImpact of dengue virus (serotype DENV-2) infection on liver of BALB/c mice: a histopathological analysis. Tissue Cell.

[66] Zagne SM (1994). Dengue haemorrhagic fever in the state of Rio de Janeiro, Brazil: a study of 56 confirmed cases. Trans. R. Soc. Trop. Med. Hyg..

[67] Mangada MN, Igarashi A (1998). Molecular and in vitro analysis of eight dengue type 2 viruses isolated from patients exhibiting different disease severities. Virology.

[68] Souza LJ (2008). Acute hepatitis due to dengue virus in a chronic hepatitis patient. Braz. J. Infect. Dis..

[69] Rasinhas AC. Estudo do Tropismo do vírus dengue tipo 4 em modelo BALB/c: infecção experimental, análises morfológicas e de viremia. Rio de Janeiro. Master’s thesis [Post-Graduation in Parasite Biology] – Instituto Oswaldo Cruz- Fiocruz; 2017.

[70] Sirivichayakul, C. *et al*. Dengue infection in children in Ratchaburi, Thailand: a cohort study. II. Clinical manifestations. *PLOS Negl. Trop. Dis.* 6(2), e1520. 10.1371/journal.pntd.0001520. (2012). 10.1371/journal.pntd.0001520 PMC3289597 22389735

[71] Hotchandani A (2014). Loss of appetite and strength in the geriatric population: diagnostic symptoms for dengue. TROP DOCT..

[72] Chen CM (2016). The outcomes of patients with severe dengue admitted to intensive care units. Medicine.

[73] Hottz ED, Bozza FA, Bozza PT (2018). Platelets in immune response to virus and immunopathology of viral infections. Front. Med..

[74] Basu A, Chaturvedi UC (2008). Vascular endothelium: the battlefield of dengue viruses. FEMS Immunol. Med. Microbiol..

[75] de Azeredo, E. L., Monteiro, R. Q., & de-Oliveira Pinto, L. M. Thrombocytopenia in Dengue: Interrelationship between virus and the imbalance between coagulation and fibrinolysis and inflammatory mediators. *Mediators Inflamm.* 2015, 313842. 10.1155/2015/313842. (2015). 10.1155/2015/313842 PMC4427128 25999666

[76] Pluthero FG, Kahr W (2018). The birth and death of platelets in health and disease. Physiology.

[77] Torres EM (2008). Dengue. Estud. av..

[78] Eu-Ahsunthornwattana, N., & Thisyakorn, U. Peripheral blood count for Dengue severity prediction: aprospective study in Thai children. *Pediatrics* 121(2), 127S128. 10.1542/peds.2007-2022KKKK. (2008).

[79] Rajmane Y (2013). Infant mouse brain passaged Dengue serotype 2 virus induces non-neurological disease with inflammatory spleen collapse in AG129 mice after splenic adaptation. Virus Res..

[80] Soni, A., Patel, PM., Malhi, N.S., & Avasth,i G.L. Spectrum of liver dysfunction in patients with dengue infection and the markers of severe disease: study from a tertiary care centre in Punjab. *J. Liver Res. Disord Ther*. 3(4), 95–8. 10.15406/jlrdt.2017.03.00063 (2017).

[81] Seneviratne SL, Malavige GN, de Silvac HJ (2006). Pathogenesis of liver involvement during dengue viral infections. Trans. R. Soc. Trop. Med. Hyg..

[82] Dalugama C, Gawarammana IB (2018). Lessons learnt from managing a case of dengue hemorrhagic fever complicated with acute liver failure and acute kidney injury: a case report. J. Med. Case Rep..

[83] Ozer J, Ratner M, Shaw M, Bailey W, Schomaker S (2008). The current state of serum biomarkers of hepatotoxicity. Toxicology.

[84] Nguyen TL, Nguyen TH, Tieu NT (1997). The impact of dengue haemorrhagic fever on liver function. Res. Virol..

[85] Ferreira R (2018). Predictive factors of dengue severity in hospitalized children and adolescents in Rio de Janeiro Brazil. Rev. Soc. Bras. Med. Trop..

[86] Singer, C., Stancu, P., Coşoveanu, S., & Botu, A. Non-alcoholic fatty liver disease in children. *Curr Health Sci J*. 40(3), 170–176. 10.12865/CHSJ.40.03.03. (2014). 10.12865/CHSJ.40.03.03 PMC4340436 25729601

[87] Bhattacharya D, Angurana SK, Nallasamy K, Iyer R, Jayashree M (2019). Severe dengue and associated hemophagocytic lymphohistiocytosis in PICU. Indian J. Pediatr..

[88] Barreto DF (2004). Histopathological aspects of Dengue-2 virus infected mice tissues and complementary virus isolation. J. Submicrosc. Cytol. Pathol..

[89] Gulati S, Maheshwari A (2007). Atypical manifestations of dengue. Trop. Med. Int. Health..

[90] Barth OM (2006). Morphological studies in a model for dengue-2 virus infection in mice. Mem. Inst. Oswaldo Cruz.

[91] Saxena R (2018). Practical Hepatic Pathology: a Diagnostic Approach.

[92] Chau TN (2004). SARS-associated viral hepatitis caused by a novel coronavirus: report of three cases. Hepatology.

[93] Sanyal AJ (2005). Review article: non-alcoholic fatty liver disease and hepatitis C - risk factors and clinical implications. Aliment Pharmacol. Ther..

[94] Randall G (2018). lipid droplet metabolism during dengue virus infection. Trends Microbiol..

[95] Samsa MM (2009). Dengue virus capsid protein usurps lipid droplets for viral particle formation. PLoS Pathog..

[96] Zhang J (2018). Flaviviruses exploit the lipid droplet protein AUP1 to trigger lipophagy and drive virus production. Cell Host Microbe.

[97] Rabelo LM (2018). Histological liver chances in Swiss mice caused by tannery effluent. Environ Sci. Pollut. Res. Int..

[98] Dumková J (2017). Sub-chronic inhalation of lead oxide nanoparticles revealed their broad distribution and tissue-specific subcellular localization in target organs. Part Fibre Toxicol..

[99] Ip YT, Dias Filho MA, Chan JK (2010). Nuclear inclusions and pseudoinclusions: friends or foes of the surgical pathologist?. Int. J. Surg. Pathol..

[100] Grizzi F, Chiriva-Internati M (2007). Human binucleate hepatocytes: are they a defence during chronic liver diseases?. Med. Hypotheses.

[101] Toyoda H (2005). Changes to hepatocyte ploidy and binuclearity profiles during human chronic viral hepatitis. Gut.

[102] Austin LS, Kaushansky A, Kappe SH (2014). Susceptibility to Plasmodium liver stage infection is altered by hepatocyte polyploidy. Cell. Microbiol..

[103] Wang MJ, Chen F, Lau J, Hu YP (2017). Hepatocyte polyploidization and its association with pathophysiological processes. Cell Death Dis..

[104] Almeida, A.L.T. Estudos Morfológicos De Tecidos De Modelo Murino Balb/C Com Quadro De Reinfecção Pelos Vírus Dengue. Rio de Janeiro. Graduation monography. [Graduation in Biological Sciences] – Universidade Federal do Rio de Janeiro; 2018.

[105] Hedgecock EM, White JG (1985). Polyploid tissues in the nematode Caenorhabditis elegans. Dev. Biol..

[106] Glotzer M (2005). The molecular requirements for cytokinesis. Science.

[107] Duelli D, Lazebnik Y (2007). Cell-to-cell fusion as a link between viruses and cancer. Nat. Rev. Cancer.

[108] Gentric G, Desdouets C (2014). Polyploidization in liver tissue. Am. J. Pathol..

[109] Miyaoka Y (2012). Hypertrophy and unconventional cell division of hepatocytes underlie liver regeneration. Curr. Biol..

[110] Puerta-Guardo, H., Glasner, D. R., & Harris, EDengue Virus NS1 disrupts the endothelial glycocalyx, leading to hyperpermeability. *PLoS Pathog*. 12(7), e1005738. 10.1371/journal.ppat.1005738. (2016). 10.1371/journal.ppat.1005738 PMC4944995 27416066

[111] Zellweger RM, Prestwood TR, Shresta S (2010). Enhanced infection of liver sinusoidal endothelial cells in a mouse model of antibody-induced severe dengue disease. Cell Host Microbe.

[112] Malavige GN, Ogg GS (2017). Pathogenesis of vascular leak in dengue virus infection. Immunology.

[113] Aye KS (2014). Pathologic highlights of dengue hemorrhagic fever in 13 autopsy cases from Myanmar. Hum pathol.

[114] Secretaria de Vigilância Sanitária/Ministério da Saúde. [https://portalarquivos2.saude.gov.br./] Brasília (DF): Boletim Epidemiológico. Volume 49 - no 7 - 2018. Monitoramento dos casos de dengue, febre de chikungunya e febre pelo vírus Zika até a Semana Epidemiológica 5 de 2018. Available in: https://antigo.saude.gov.br/images/pdf/2018/fevereiro/20/2018-007.pdf. Accessed: 12 Aug 2020.

[115] Secretaria de Vigilância Sanitária/Ministério da Saúde. [https://portalarquivos2.saude.gov.br./] Brasília (DF):. Boletim Epidemiológico. Volume 51 - no 31 - 2020. Monitoramento dos casos de arboviroses urbanas transmitidas pelo Aedes Aegypti (dengue, chikungunya e zika), semanas epidemiológicas 1 a 29, 2020. Available in: https://antigo.saude.gov.br/.images/pdf/2020/August/06/Boletim-epidemiologico-SVS-31.pdf. Accessed: 12 Aug 2020.

[116] Gubler DJ, Kuno G, Sather GE, Velez M, Oliver A (1984). Mosquito cell cultures and specific monoclonal antibodies in surveillance for dengue viruses. Am. J. Trop. Med. Hyg..

[117] Lanciotti RS, Calisher CH, Gubler DJ, Chang GJ, Vorndam AV (1992). Rapid detection and typing of dengue viruses from clinical samples by using reverse transcriptase-polymerase chain reaction. J. Clin. Microbiol..

[118] Reed LJ, Muench H (1938). A simple method of estimating fifty percent endpoints. Am. J. Epidemiol..

